# 181. Impact of Rapid Diagnostic Technology on Patients with Candidemia

**DOI:** 10.1093/ofid/ofac492.259

**Published:** 2022-12-15

**Authors:** Patrick Funderburk, Amy L Carr, Jillian E Hayes

**Affiliations:** AdventHealth Orlando, Orlando, Florida; AdventHealth Orlando, Orlando, Florida; Duke University Hospital, Durham, North Carolina

## Abstract

**Background:**

Candida spp. are the fourth most common cause of bloodstream infections (BSI) in the United States and have an associated mortality rate of 19-40.5%. Mortality rates are further impacted by delay in time to adequate antifungal therapy, historically due to delayed time to identification on culture. The utilization of rapid diagnostic technology (RDT) has been effective in timely identification of bacterial pathogens causing BSI, but RDT for fungal organisms has demonstrated mixed results. At AdventHealth Central Florida Division South (CFD-S), pharmacists provide 24-hour coverage for real-time notification of all positive blood culture results. The objective of this study was to evaluate the clinical impact of the GenMark ePlex Blood Culture Identification Panels (BCID) fungal pathogen (FP) panel paired with 24/7, pharmacist-driven response in patients with candidemia.

**Methods:**

This multi-site, pre/post, retrospective chart review included adult patients admitted to CFD-S with at least one positive blood culture with Candida spp. from June 2019 through May 2020 (pre-RDT), and August 2020 through July 2021 (post-RDT). Patients receiving systemic antifungal prophylaxis, with known candidiasis at time of index RDT result, or who discharged prior to culture positivity were excluded. The primary outcome was time to effective antifungal therapy in patients with candidemia.

**Results:**

A total of 200 patients were included in the study (100 pre-RDT and 100 post-RDT). Overall, patients had a median age of 61 years and 50% were male. Patient characteristics are summarized in Table 1; median APACHEII score differed by three points (13.5 vs. 16.5). Time to effective therapy was similar between groups (39.8 vs. 38.5 hours, p=0.217). There was no difference in secondary outcomes (Table 2) other than ICU length of stay (2.5 vs. 6.0 days, p=0.033) and all-cause in-hospital mortality (15% vs. 30%, p=0.011).

**Figure:**
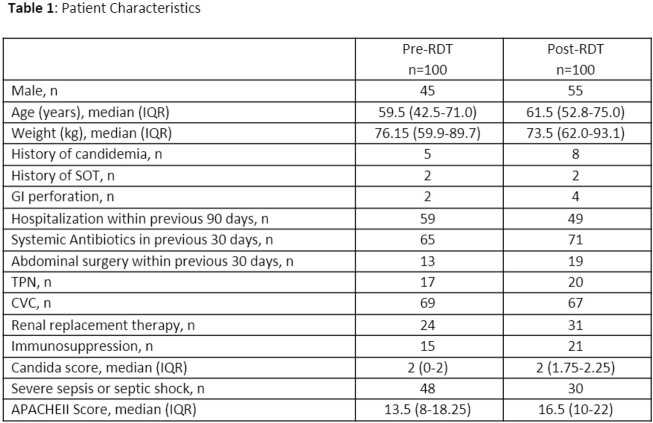

**Figure:**
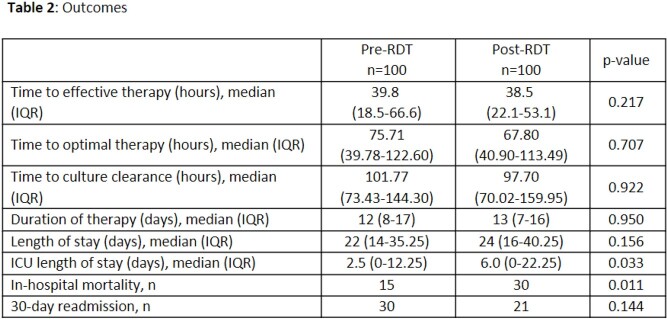

**Conclusion:**

Pharmacist-driven, real-time response to RDT did not significantly impact time to effective antifungal therapy in patients with candidemia. Higher rate of in-hospital mortality was likely a reflection of increased severity of illness in the post-RDT group.

**Disclosures:**

**Amy L. Carr, PharmD, BCIDP**, Shionogi: Advisory Board.

